# APP-BACE1 Interaction and Intracellular Localization Regulate Aβ Production in iPSC-Derived Cortical Neurons

**DOI:** 10.1007/s10571-023-01374-0

**Published:** 2023-06-24

**Authors:** Sandra Roselli, Tugce Munise Satir, Rafael Camacho, Stefanie Fruhwürth, Petra Bergström, Henrik Zetterberg, Lotta Agholme

**Affiliations:** 1 Institute of Neuroscience and Physiology, Department of Psychiatry and Neurochemistry, The Sahlgrenska Academy at the University of Gothenburg, Blå Stråket 15, Sahlgrenska Hospital, 405 30 Gothenburg, Sweden; 2 Centre for Cellular Imaging, Core Facilities, The Sahlgrenska Academy, University of Gothenburg, Medicinaregatan 7A, 405 30 Gothenburg, Sweden; 3 Clinical Neurochemistry Laboratory, Sahlgrenska University Hospital, Building V3, Mölndal Hospital, 431 80 Mölndal, Sweden; 4 Department of Neurodegenerative Disease, Institute of Neurology, University College London Queen Square, Queen Square, London, WC1N 3BG UK; 5 UK Dementia Research Institute at UCL, Cruciform Building, Gower Street, London, WC1E 6BT UK; 6 Hong Kong Center for Neurodegenerative Diseases, Units 1501-1502, 1512-1518, 15/F, Building 17W, Hong Kong Science Park, Shatin, N.T., Hong Kong, China; 7 Wisconsin Alzheimer’s Disease Research Center, University of Wisconsin School of Medicine and Public Health, University of Wisconsin-Madison, 600 Highland Avenue, Madison, WI 53792 USA

**Keywords:** Amyloid precursor protein, Beta secretase, Gamma secretase, Amyloid beta, Neurons, Proximity ligation assay, Early endosomes

## Abstract

Alzheimer’s disease (AD) is characterized pathologically by amyloid β (Aβ)-containing plaques. Generation of Aβ from amyloid precursor protein (APP) by two enzymes, β- and γ-secretase, has therefore been in the AD research spotlight for decades. Despite this, how the physical interaction of APP with the secretases influences APP processing is not fully understood. Herein, we compared two genetically identical human iPSC-derived neuronal cell types: low Aβ-secreting neuroprogenitor cells (NPCs) and high Aβ-secreting mature neurons, as models of low versus high Aβ production. We investigated levels of substrate, enzymes and products of APP amyloidogenic processing and correlated them with the proximity of APP to β- and γ-secretase in endo-lysosomal organelles. In mature neurons, increased colocalization of full-length APP with the β-secretase BACE1 correlated with increased β-cleavage product sAPPβ. Increased flAPP/BACE1 colocalization was mainly found in early endosomes. In the same way, increased colocalization of APP-derived C-terminal fragment (CTF) with presenilin-1 (PSEN1), the catalytic subunit of γ-secretase, was seen in neurons as compared to NPCs. Furthermore, most of the interaction of APP with BACE1 in low Aβ-secreting NPCs seemed to derive from CTF, the remaining APP part after BACE1 cleavage, indicating a possible novel product-enzyme inhibition. In conclusion, our results suggest that interaction of APP and APP cleavage products with their secretases can regulate Aβ production both positively and negatively. β- and γ-Secretases are difficult targets for AD treatment due to their ubiquitous nature and wide range of substrates. Therefore, targeting APP-secretase interactions could be a novel treatment strategy for AD.

**Graphical Abstract:**

Colocalization of APP species with BACE1 in a novel model of low- versus high-Aβ secretion—Two genetically identical human iPSC-derived neuronal cell types: low Aβ-secreting neuroprogenitor cells (NPCs) and high Aβ secreting mature neurons, were compared. Increased full-length APP (flAPP)/BACE1 colocalization in early endosomes was seen in neurons, while APP-CTF/BACE1 colocalization was much higher than flAPP/BACE1 colocalization in NPCs, although the cellular location was not determined.

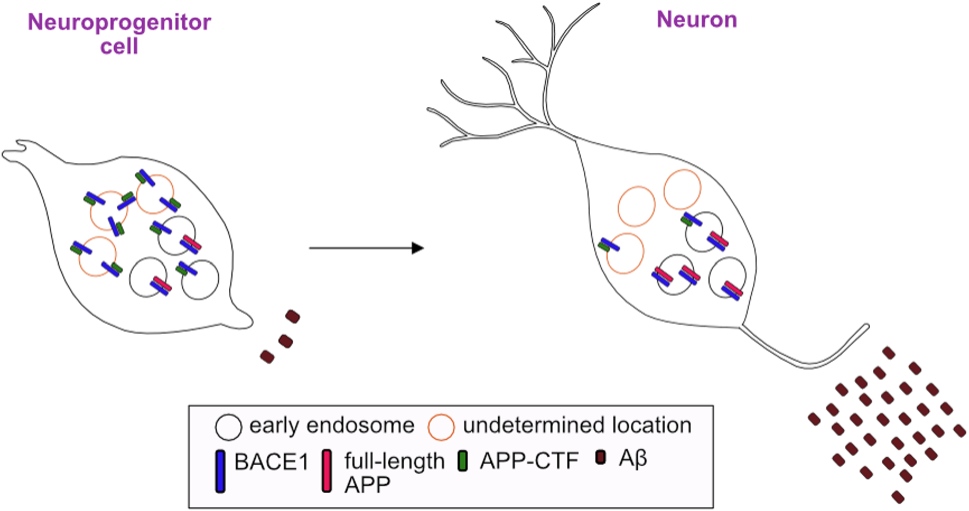

**Supplementary Information:**

The online version contains supplementary material available at 10.1007/s10571-023-01374-0.

## Introduction

**Fig. 1:**
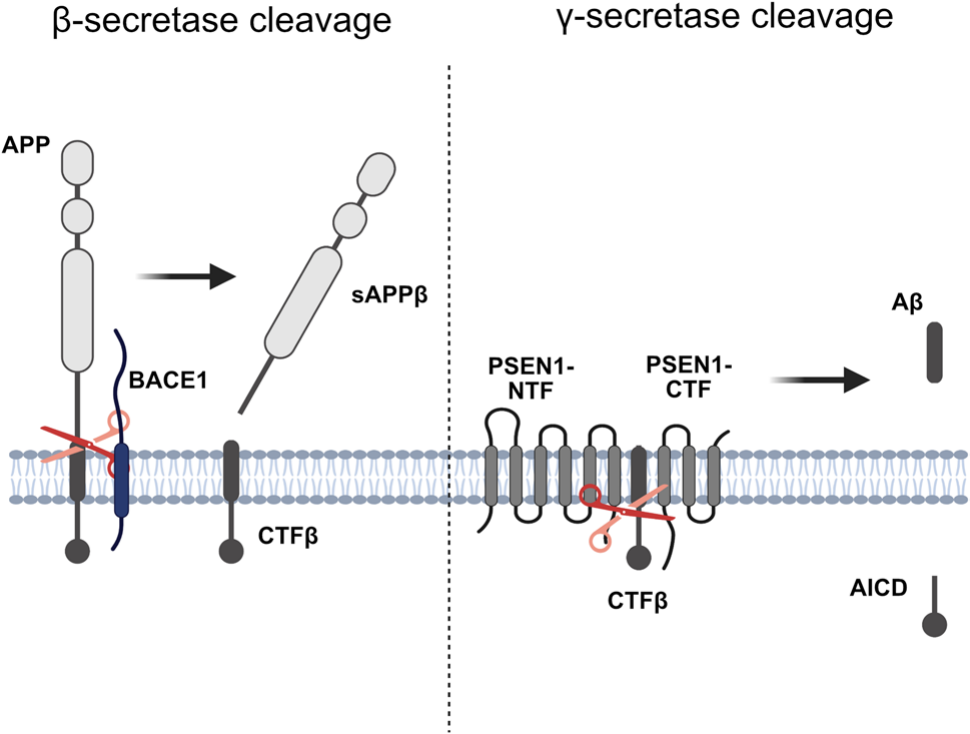
Schematic illustration of the amyloidogenic pathway of APP cleavage. Amyloid precursor protein (APP) is initially cleaved by BACE1 (β-secretase), releasing soluble APPβ (sAPPβ) and leaving membrane-anchored C-terminal fragment β (CTFβ). CTFβ is then further cleaved by the heterodimer of presenilin 1 fragments (PSEN1-NTF and PSEN1-CTF) in the γ-secretase complex, releasing Aβ peptides extracellularly or intra-vesicular, and amyloid intracellular domain (AICD) into the cytosol

Alzheimer’s disease (AD) is the most common form of dementia, characterized by progressive cognitive decline due to neurodegeneration. One of the major neuropathological hallmarks of AD is the accumulation of amyloid β (Aβ)-containing plaques (Blennow et al. 2006). Aβ peptides are generated from sequential enzymatic cleavages of amyloid precursor protein (APP) (Portelius et al. 2011). Mutations in *APP* that alter the processing of its gene product, or in *PSEN1/2*, coding for the active subunit of the cleaving enzyme γ-secretase, give rise to the familial form of early-onset AD (Bekris et al. 2010). The mechanism and regulation of APP cleavage into Aβ have therefore been in the spotlight of AD research for decades. APP is a single-pass transmembrane protein, with a long N-terminal ectodomain and a short C-terminal part on the cytosolic side that undergoes substantial post-translational proteolytic processing. This occurs through two main pathways: the non-amyloidogenic pathway, which prevents full-length Aβ production (Mattson et al. 1993) through initial cleavage of APP by α-secretase inside the Aβ domain (Portelius et al. 2011), and the amyloidogenic pathway, which leads to the generation of full-length Aβ (mainly 38–42 amino acids long) (Walsh and Selkoe 2007). Through the amyloidogenic pathway, APP is initially cleaved by β-secretase, generating a short membrane-bound C-terminal fragment β (CTFβ) including the entire Aβ domain, and a long soluble APPβ fragment (sAPPβ). CTFβ, a direct precursor of Aβ, is then cleaved further by γ-secretase into Aβ peptides that are secreted to the extracellular space and into the cerebrospinal fluid (Perreau et al. 2010; Seubert et al. 1992). The amyloidogenic pathway is summarized in Fig. 1.

BACE1 is the main enzyme in neurons performing the β-cleavage of APP that gives rise to amyloidogenic peptides (Vassar et al. 1999), and hence, BACE1 cleavage of APP is considered the initial step for Aβ generation (Vassar 2004). APP β-cleavage is believed to happen mainly in early and recycling endosomes, and APP endocytosed from the plasma membrane was shown to account for most of Aβ production (Haass et al. 2012; Ehehalt et al. 2003; Kinoshita et al. 2003; Carey et al. 2005; Cirrito et al. 2008). Interestingly, although both APP and BACE1 are produced and matured in the secretory pathway, newly synthesized APP and BACE segregate into two distinct ER- and trans-Golgi-derived vesicles, suggesting that APP/BACE1 interaction is not left to chance, but rather tightly regulated (Toh et al. 2017, 2018; Tan et al. 2020; Fourriere et al. 2022).γ-Secretase is a multi-protein protease complex consisting of four subunits, where either presenilin-1 (PSEN1) or PSEN2 is the catalytic subunit in amyloidogenic Aβ production (Kimberly et al. 2000). PSEN1 is synthesized as a ~ 50 kDa precursor, that gets cleaved into a ~ 30 kDa N-terminal fragment (NTF) and a ~ 20 kDa C-terminal fragment (CTF) during maturation. Only PSEN1-NTF and CTF heterodimers are catalytically active (Ward et al. 1996; Dries and Yu 2008). Nicastrin, another of the four subunits of the γ-secretase complex, acts as a gatekeeper for substrate entry and only allows small substrates to reach the active part of the γ-secretase complex (De Strooper 2005). Thus, bigger proteins such as APP need to be processed through ectodomain-shedding prior to γ-secretase cleavage (Bolduc et al. 2016). Since all four components of γ-secretase are found in several locations inside the cell (Cupers et al. 2001; Siman and Velji 2003), the exact site of γ-cleavage has been challenging to assess and it is still under investigation. However, it has been suggested that proteolytically active γ-secretase complex is localized in the plasma membrane and the endosomal/lysosomal system (Fukumori et al. 2006; Dries and Yu 2008; Maesako et al. 2022). β- and γ-Secretase are ubiquitous enzymes that are responsible for the cleavage of several substrates in neurons and other cells (Kopan and Ilagan 2004; Yan 2017; Das et al. 2016), making them difficult therapeutic targets to reduce amyloid generation and deposition in the AD brain. Alternatively, the molecular interaction of APP with secretases could be blocked, sparing off-target effects. Therefore, shedding light on the mechanisms of interaction between APP and the cleaving enzymes upon production of Aβ could be important for the development of effective preventive or therapeutic treatments.

We have previously shown that APP processing along the β-/γ-dependent pathway, producing amyloidogenic Aβ peptides, increases gradually during cortical neuronal differentiation (Bergstrom et al. 2016; Satir et al. 2020) from induced pluripotent stem cells (iPSCs). Here we take advantage of the physiological difference in APP amyloidogenic processing between low Aβ-secreting neural progenitor cells (NPCs) and high Aβ-secreting neurons, to determine how secretion of Aβ is regulated, based on the expression and colocalization of APP and its secretases.

## Material and Methods

### Cell Culture

Human iPSC lines, from healthy individuals, WTSLIi015-A (EBiSC via Sigma Aldrich) and ChiP22 (Takara Bio) were cultured on hESC qualified matrigel (Corning) in mTeSR1 media (Stemcell technologies). Media change was performed every day and cells were passaged upon 80–100% confluency with 0.5 mM EDTA (Thermo Fisher Scientific). iPSCs from healthy individuals were differentiated towards cortical neurons according to Shi et al. (2012), with slight modifications (Bergstrom et al. 2016). On day 35 of differentiation, NPCs were seeded onto poly-L-ornithine (0.01%; Sigma Aldrich) and laminin (1–2 μg/cm^2^; Sigma Aldrich)-coated plates in neuronal maintenance media and maintained for 10 or 40 days with media change every second day. Media was supplemented with 0.5–1 μg/cm^2^ laminin every 10th day.

SH-SY5Y cells (ECACC) were cultured in DMEM/F-12 + Glutamax (Thermo Fisher Scientific) supplemented with 10% fetal calf serum (FCS; Sigma Aldrich) and 100 U/ml penicillin/streptomycin (GE Healthcare). All cells were cultured in a humified environment at 37 ℃, at 5% CO_2_ and ambient O_2_.

### APP Knockdown in SH-SY5Y Cells

One day before transfection, cells were dissociated with 0.25% trypsin/EDTA (Thermo Fisher Scientific) and re-seeded in antibiotic-free media at a density of 85,000 cells/cm^2^ onto glass coverslips placed in a 6 well plate format. The following day, cells were transfected with 25 pmol/well of either negative control siRNA (Ambion #4390843) or APP siRNA (Ambion #4427038) using Lipofectamine RNAiMAX Transfection Reagent (all from Thermo Fisher Scientific) according to the manufacturer’s instruction. Cells were incubated for 48 h and re-transfected following the same procedure. Twenty-four hours after the second transfection, cells on coverslips were washed once in PBS, fixed in Histofix (Histolab Products) for 15 min at room temperature, and stored in PBS at 4 degrees until immunostaining. The remaining cells in the wells were collected for quantification of APP using western blot.

### Transduction with BacMam GFP-CellLight®

After about 45 or 75 days in culture, iPSC-derived NPCs or neurons were transduced with GFP-CellLight® (Thermo Fisher Scientific) for early endosomes (Rab5a), late endosomes (Rab7a) and lysosomes (Lamp1) markers using BacMam delivery technology. The amount of the virus was calculated according to manufacturer´s recommendations. For early endosomes, 30 virus particles per cell and for late endosomes and lysosomes, 45 virus particles per cell were used. Cells were incubated overnight at 37 ℃, at 5% CO_2_ and ambient O_2_ and fixed the next day.

### Quantification of sAPPβ, CTFβ and Aβ Peptides

For secreted  protein fragments, cell-conditioned culture media were collected after 48 h of incubation, centrifuged at 400 g to remove cell debris, and stored at − 80 ℃ until further analysis. For intracellular CTFβ and Aβ peptides, cells were washed once in PBS, collected in PBS, centrifuged, and stored at − 80 ℃ until further analysis. sAPPβ concentrations in cell-conditioned media were analyzed using the MSD sAPPα/sAPPβ Multiplex Assay, as described by the manufacturer (Meso Scale Disovery). Aβx-38/40/42 concentrations in cell-conditioned media and intracellular Aβx-38/40/42 concentrations were measured using the MSD Human (6E10) Aβ Triplex Assay as described by the manufacturer (Meso Scale Discovery). For intracellular Aβ, cells were lysed in RIPA buffer, as described previously (Bergstrom et al. 2016) and protein concentration was determined using the Pierce BCA protein assay kit (Thermo Fisher Scientific), according to the manufacturers’ protocol. For each cell lysate sample, 70 µg of total protein was loaded in the MSD plate. Standard peptides were diluted in RIPA buffer supplemented with 1% BSA. The limit of detection was set to the value of the lowest standard point. All samples with measurements below detection limit were assigned a fixed concentration corresponding to half of the detection limit. Assay plates were read by using QuickPlex SQ 120 instrument and analyzed by Discovery Workbench software (Meso Scale Discovery). Intracellular CTFβ was measured with a human APP βCTF Assay Kit (IBL, catalog number 27776) as described by the manufacturer. Briefly, cells were lysed in MSD lysis buffer (150 mM NaCl, 20 mM Tris, pH 7.5, 1 mM EDTA, 1 mM EGTA, 1% Triton X-100) supplemented with protease inhibitor cocktail (Roche). Total protein concentrations were determined by using the Pierce BCA protein assay kit (Thermo Fisher Scientific) according to the manufacturer´s instruction. Twenty µg of total protein was used for each sample. The absorbance measurements were performed using Infinite F50 Tecan plate reader instrument with Magellan™ reader control and data analysis software.

### Immunocytochemistry

Cells cultured on Ibidi μ-slides (Ibidi) were fixed with 4% paraformaldehyde (PFA) for 10 min at room temperature and then washed 3 × 5 min with TBS (Medicago). Cells were permeabilized by incubation in 0.3% Triton-X100 (Sigma Aldrich) in TBS for 15 min at room temperature and thereafter incubated in blocking buffer (5% donkey serum (Sigma Aldrich), 0.3% Triton-X100 in TBS) for one hour at room temperature. Primary antibodies were diluted to the appropriate dilutions (see Supplementary Table 1) in blocking buffer and incubated at 4 °C over night or for 1.5 h in room temperature. After washing with TBS, samples were incubated with appropriate Alexa conjugated secondary antibodies (Thermo Fisher Scientific), diluted in blocking buffer (1:400), for one hour at room temperature. Samples were then washed 3 × 5 min with TBS, and DAPI was added in the second wash. Samples were then washed once with H_2_O and thereafter mounted in Ibidi mounting medium (Ibidi).

### Proximity Ligation Assay

Cells cultured on Ibidi μ-slides (Ibidi) were fixed and blocked as described previously. Primary antibodies against APP, PSEN1 and BACE1 were diluted to the appropriate concentrations (see Supplementary Table 1) in blocking buffer and samples were incubated over night at 4 °C. Samples were washed with DuoLink Wash Buffer A, and thereafter incubated with DuoLink probe-conjugated secondary antibodies for one hour at 37 °C. After washing with Wash Buffer A, samples were incubated with ligation mix for 30 min at 37 °C. Thereafter, samples were washed once again with DuoLink Wash Buffer A, followed by incubation with amplification solution for 100 min at 37 °C, for ligated oligonucleotides to be amplified. After the amplification step, samples were kept in dark and the remaining wash steps were performed according to the manufacturer’s instructions using DuoLink wash buffers. The samples were thereafter dried and mounted using DuoLink mounting media with DAPI (all DuoLink products from Sigma Aldrich). Samples were stored at − 20 °C until further analysis. Neurons incubated with APP C-terminal primary antibody only and both secondary antibodies (mouse and rabbit) were included as a technical control (Supplementary Fig. 1A).

### Microscopy and Image Analysis

Analysis of immunocytochemistry-stained cells was performed using a Nikon Eclipse Ti-E inverted confocal microscope with 60 × objective and the NIS Element software (Nikon). *Z* stacks of images with 0.5 µm distance were acquired.

Analysis of colocalization of APP and the secretases by PLA and analysis of intracellular localization of PLA dots was performed using the Carl Zeiss LSM 880 AiryScan confocal microscope with 63 × objective and the Zen Black 2.3 software (Zeiss), within the AiryScan super-resolution mode. From each sample, images were captured at 10–15 randomly selected positions and *Z* stacks were acquired with 0.5 µm of distances between images.

In the PLA, each dot represents a colocalization event between two proteins of interest. Therefore, to quantify the colocalization of APP with secretases in NPCs and neurons we calculated the area of PLA dots and divided it by the area of nuclei. Image analysis was performed using a custom-made macro for ImageJ (Schneider et al. 2012). PLA dots were detected based on fluorescence intensity and size. DAPI-stained nuclei were detected based on fluorescence and size. The microscopy data consisted of image stacks with 3 spatial dimensions and 2 colour channels, one for nuclear staining and another for the PLA signal. Each colour channel was treated independently from each other. As a pre-processing step we applied a 3D Gaussian blur (sigma = 3 and 1 for Nucleus and PLA signal, respectively). Then the z index of best focus was calculated based on the average intensity of each image plane. To find the lower and upper threshold values for the Nucleus channel we implemented Li’s Minimum Cross Entropy thresholding method to the image of best focus. On the other hand, to find the lower and upper threshold values for the PLA channel we implemented the Triangle thresholding method to the image of best focus. After applying the threshold to the 3D stacks of both colour channels, we calculated the ratio of the PLA area to Nucleus area.

To investigate the intracellular localization of APP/secretases colocalization, we calculated the percentage of organelle (GFP-tagged organelle marker) that was occupied by PLA dots. Five to fifteen images with GFP-organelle positive cells were captured from each sample, and z stacks were acquired with 0.5 µm of distance between images, and 21 images per stack. Image analysis was performed using a second custom-made macro for ImageJ. The microscopy data consisted of image stacks with 3 spatial dimensions and 2 colour channels, one for Organelle staining and another for PLA. Each colour channel was treated independently from each other. Initially, the z-index of best focus for each colour channel was found based on the average intensity of each image plane. To find the lower and upper threshold values for both Organelle and PLA channels we implemented the Triangle thresholding method to the image of best focus. After applying the threshold to the 3D stacks of both colour channels, the binary colocalization between them was calculated. Finally, we calculated the percentage of the PLA/Organelle colocalization area to Organelle area.

### Western Blot

Cells were lysed as described for quantification of intracellular Aβ. For each sample, 10 µg of total protein was mixed with NuPAGE 4 × LDS Sample Buffer, and dithiothreitol (DTT; 50 mM), boiled at 95 °C for 5 min and loaded onto a 4–12% NuPAGE) Bis–Tris gel and run with MES buffer (all from Thermo Fisher Scientific). Using semi-dry technique, the gels were blotted onto 0.2 µM nitrocellulose membranes. The membranes were blocked in 5% non-fat dry milk (BioRad laboratories) for 1 h and incubated with primary antibodies diluted in blocking buffer over night at 4 °C (see Supplementary Table 1 for antibodies and dilutions). Membranes were washed in TBS with 0.05% Tween (Sigma Aldrich) and incubated with HRP-conjugated anti-mouse or anti-rabbit secondary antibodies (1:2000, Cell Signaling Technology) in blocking solution for 1 h at room temperature. SuperSignal West Dura Extended Duration Substrate (Thermo Fisher Scientific) was used to develop the membranes and bands were visualized using ChemiDoc XRS + (BioRad laboratories). The membrane was stripped using Restore stripping buffer (Thermo Fisher Scientific), blocked as described above, and then re-incubated with an HRP-conjugated glyceraldehyde-3-phosphate dehydrogenase (GAPDH) antibody (see Supplementary Table 1 for antibodies and dilutions) diluted in blocking solution at room temperature for 1 h. Band intensities were calculated using Image Lab (BioRad laboratories) and correlated to GAPDH. Full-length blots of the proteins are presented in Supplementary Fig. 2.

### RNA Extraction and cDNA Synthesis

Cells were lysed directly in the well by addition of 600 µL Buffer RLT supplemented with 4 mM DTT (Sigma Aldrich). Total RNA was extracted with a QiaCube robotic workstation (QIAGEN), using the RNeasy Mini protocol according to manufacturer’s instructions. NanoDrop 2000/2000c spectrophotometer (Thermo Fisher Scientific) was used to measure the RNA concentrations. The total RNA was diluted to a final concentration of 25–50 ng/µL with RNase-free water. 250–500 ng of total RNA was used to synthesize cDNA with a High Capacity cDNA kit with RNase inhibitor (Applied Biosystems) and converted in a single-cycle reaction on a 2720 Thermal Cycler (Applied Biosystems); 25 °C for 10 min, 37 °C for 120 min and 85 °C for 5 min.

### Quantitative PCR

Quantitative PCR was performed using inventoried TaqMan Gene Expression Assays with FAM reporter dye in TaqMan Universal PCR Master Mix with UNG according to manufacturer’s instructions, in a total reaction volume of 25 µL. qPCR reactions were performed on Micro-Amp 96-well optical microtiter plates on a 7900HT Fast QPCR System (Thermo Fisher Scientific), using standard settings for Standard Curve qPCR. TaqMan Gene Expression Assays (all from Thermo Fisher Scientific) are listed in Supplementary Table 2. 2.5 ng of cDNA was used in the PCR and all samples were run in duplicates. PCR results were analysed with the SDS 2.3 software (Applied Biosystems) and the relative quantity of gene expression was determined using the ∆∆CT method (Livak and Schmittgen 2001), with NPCs as the calibrator and average CT:s of RPL27 and HPRT1 as endogenous reference.

### Statistics

Mean values from the separate experiments were compared using Student’s two-tailed *t* test. Statistical significance was defined as *p* < 0.05. For PLA analysis and organelle colocalization analysis, ROUT test was used to remove definite outliers, with *Q* set at 0.1%, prior to Student’s two-tailed *t* test. All statistical analyses were performed using GraphPad (Prism version 7.02 for Windows, GraphPad Software, La Jolla California USA, www.graphpad.com).

## Results

### A Physiological Difference in amyloid β secretion between Neuroprogenitor Cells and Neurons from the Same Genetic Background

**Fig. 2:**
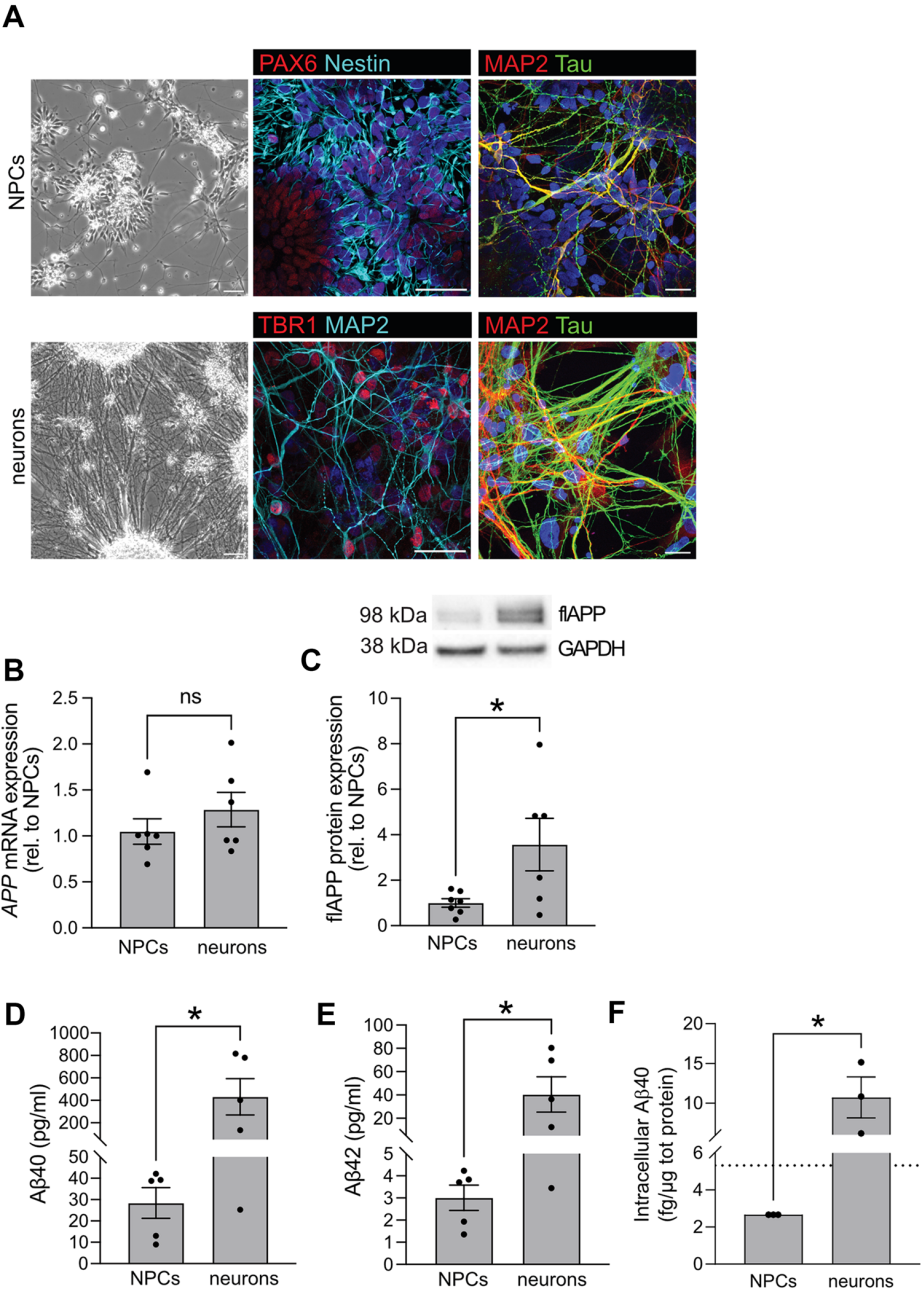
Comparison of neuronal marker expression, APP expression and amyloid β production between NPCs and neurons. **A** Visualization and characterization of neuroprogenitor cells (NPCs) and neurons. Phase contrast images of NPCs, cultured for 45 days, showed cells with small neurite extensions (upper left panel). NPCs stained positive for the neuronal progenitor markers PAX6 and nestin (upper middle panel). NPCs stained weakly for the neuronal structure markers, MAP2 and tau (upper right panel). Further differentiation, for a total of 75 days, resulted in mature neurons with extensive neurite outgrowth and networks (bottom left panel). These cells stained positive for the cortical marker TBR1 and dendritic marker MAP2 (bottom middle panel) and displayed strong staining of neuronal structure proteins tau and MAP2 (bottom right panel). Scale bar in left panels = 50 μm, in middle and right panels 25 μm. **B** mRNA expression of *APP* was investigated by qPCR. No significant difference in *APP* mRNA was found in NPCs as compared to neurons Bars represent mean of three separate differentiations from two different iPSC lines ± SEM. **C** Protein expression of APP was investigated by western blot. APP protein expression was increased in neurons as compared to NPCs. Full-length blots are available in supplementary Fig. 2. Bars represent mean of six separate differentiations from two different iPSC lines ± SEM. *p < 0.05. **D**–**F** Secreted and intracellular levels of Aβ40 and Aβ42 were investigated using an immunoassay with electrochemoluminescence detection. Secretion of both Aβ40 (**D**) and Aβ42 (**E**) was increased more than tenfold in neurons as compared to NPCs. Bars represent mean of five separate differentiations from two different iPSC lines ± SEM. Intracellular Aβ40 was also significantly increased in neurons (**F**). The dotted line represents the detection limit. Bars represent mean of three separate differentiations from two different iPSC lines ± SEM

Human iPSCs were differentiated for 45 days, into NPCs, or for 75 days, into post-mitotic neurons according to previously published protocols (Shi et al. 2012; Bergstrom et al. 2016). The two time points were chosen due to their previously reported differences in Aβ secretion (Bergstrom et al. 2016). Morphological assessment showed that NPCs displayed short neurites, and stained positive for the neuronal progenitor markers PAX6 and nestin, whereas staining for neuronal structure proteins MAP2 and tau was weak (Fig. 2A upper panels). Neurons displayed long neurites and a connected neurite network. These cells stained positive for the cortical marker TBR1, and displayed strong staining for the neuronal structure proteins MAP2 and tau (Fig. 2A lower panels). No difference in *APP* mRNA expression was found (Fig. 2B), but APP protein expression was about 3 times higher in neurons as compared to NPCs (Fig. 2C).

As previously reported (Bergstrom et al. 2016), the secretion of Aβ40 (Fig. 2D) and Aβ42 (Fig. 2E) was low in NPCs and increased more than tenfold as the cells matured into cortical neurons. Levels of intracellular Aβ40 were also significantly increased in neurons (Fig. 2F), suggesting an overall increased production of amyloidogenic Aβ species upon neuronal maturation. We took advantage of this physiological difference in APP amyloidogenic processing in the two cell types, and studied the driving molecular event for Aβ production; that is colocalization of APP with the cleaving enzymes.

### Colocalization of BACE1 with Full-Length APP, but not C-Terminal APP Fragment, in Early Endosomes Correlates with APP β-Cleavage

**Fig. 3:**
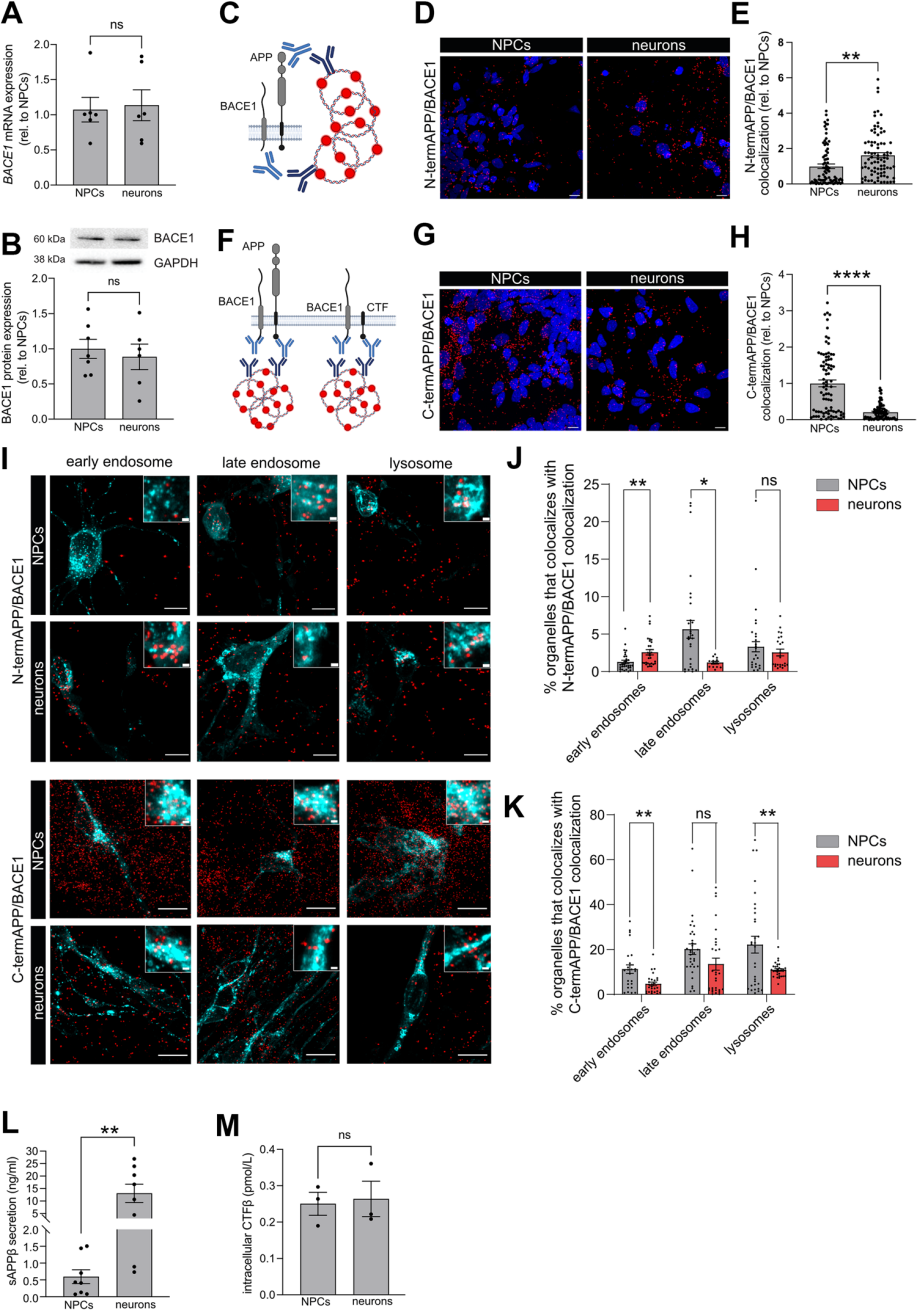
Colocalization of BACE1 with N-terminal APP, but not C-terminal APP, in early endosomes correlates with APP β-cleavage.** A** mRNA expression of *BACE1* was investigated by qPCR. No difference in *BACE1* mRNA expression was observed between NPCs and neurons. Bars represent mean of three separate differentiations from two different iPSC lines ± SEM. **B** Protein expression of BACE1 was investigated by western blot. Expression of BACE1 protein did not differ between NPCs and neurons. Full-length blots are available in Supplementary Fig. 2. Bars represent mean of six separate differentiations from two different iPSC lines ± SEM. **C**–**E** A proximity ligation-based immunocytochemistry assay was used to investigate the colocalization of BACE1 with N-terminal APP. **C** A schematic image of the binding sites for the oligonucleotide-tagged antibodies detecting BACE1 and flAPP. **D** Colocalization, visualized as red dots, between BACE1 and N-terminal APP was increased in neurons as compared to NPCs. Scale bar = 10 μM. **E** A significant increase in colocalization signal per cell was found in neurons as compared to NPCs. Data are presented as fold change related to NPCs, set to 1 and bars represent mean + / − SEM. 81 (NPC) or 83 (neurons) images from four to five separate experiments on cells from at least two separate differentiations were analysed. **F**–**H** A proximity ligation-based immunocytochemistry assay was used to investigate the colocalization of BACE1 with C-terminal APP. **F** Schematic image of the binding sites for the oligonucleotide-tagged antibodies detecting BACE1 and C-terminal part of APP, enabling detection of both flAPP and APP C-terminal fragment (CTF). **G** Colocalization, visualized as red dots, between BACE1 and C-terminal APP was higher in NPCs as compared to neurons. Scale bar = 10 μm. **H** A significant increase in colocalization signal per cell was found in NPCs as compared to neurons. Data are presented as fold change related to NPCs, set to 1 and bars represent mean + / − SEM. 84 (NPC) or 77 (neurons) images from nine to eleven separate experiments on cells from three to four separate differentiations from two different iPSCs lines were analysed. **I** Signal overlap between APP/BACE1 PLA (red) and GFP-tagged organelle markers for early endosomes (Rab5a) late endosomes (Rab7a) and lysosomes (lamp1) (cyan) was used to determine the intracellular localization of APP/BACE1 colocalization. Smaller boxes show a zoom-in of organelle colocalization with PLA signals. Scale bar = 10 μm (large boxes), 1 μm (small boxes). A significant increase in early endosomes and decrease in late endosomes colocalization with N-termAPP/BACE1 (**J**) and a decrease in all organelle colocalization with C-termAPP/BACE1 (**K**) was found in neurons as compared to NPCs. Data are presented as percentage of total organelle area and bars represent mean + / − SEM. 13–41 images for each organelle from three separate experiments on cells from two different iPSCs lines were analysed. **L** Secretion of the N-terminal soluble part of APP (sAPPβ) was investigated using electrochemiluminescence. The secretion of sAPPβ was increased about 20-fold in neurons as compared to NPCs. Bars represent mean of eight separate differentiations from four different iPSC lines ± SEM. M) Intracellular levels of CTFβ was investigated using ELISA. Levels of CTFβ did not differ between NPCs and neurons. Bars represent mean of three separate differentiations from two different iPSC lines ± SEM. All means were compared using Student’s *t* test. *p ≤ 0.05, **p ≤ 0.01, ***p ≤ 0.001, ****p ≤ 0.0001

Cleavage of APP by β-secretase is the first and rate-limiting step in the generation of Aβ. Therefore, we first investigated if the expression of BACE1, the major neuronal isoform of β-secretase, differed between NPCs and neurons. We found no difference in BACE1 expression, either on mRNA or protein levels, between the two cell types (Fig. 3A–B).

Colocalization of APP and BACE, and their localization in the organelles where BACE has its highest enzymatic activity, is fundamental for the production and secretion of Aβ (Das et al. 2016). Therefore, we investigated the colocalization of BACE1 with APP in NPCs and neurons using immunocytochemistry combined with a proximity ligation assay (PLA). In the PLA method, which utilizes short-oligonucleotide-tagged antibodies, the proximity of two target proteins (maximum 40 nm) results in the generation of fluorescent circular DNAs. These fluorescent dots were detected by confocal microscopy. Colocalization was normalized to the number of cells by calculating the ratio between the area of PLA dots and the area of nuclei (see methods for details). We first investigated colocalization of BACE1 with the substrate, full-length APP (flAPP), using an antibody directed towards the N-terminal part of APP (Fig. 3C). As expected, the colocalization of BACE1 with flAPP was significantly increased in neurons as compared to NPCs (Fig. 3D–E), suggesting that higher enzyme–substrate colocalization results in higher cleavage rate. To further investigate the colocalization of BACE1 with APP, we performed the same PLA, using an antibody directed to the C-terminal of APP. The PLA signal can then come from BACE1 interacting with flAPP as well as the C-terminal fragment of APP (Fig. 3F). Colocalization of BACE1 with C-terminal APP was highest in NPCs and decreased by almost 80% in mature neurons (Fig. 3G–H). To ensure that the PLA method detects APP-BACE colocalization specifically, we also analysed the PLA signal using C-terminal antibody for APP in SH-SY5Y cells, and found that knockdown of APP using siRNA decreased the colocalization signal (Supplementary Fig. 1B).

 β-site cleavage of APP by BACE1 is known to occur within endosomes as BACE1 needs a mildly acidic environment to function (Vassar et al. 1999). Therefore, we investigated the subcellular localization of APP with BACE1. Prior to PLA, cells were transduced with GFP-tagged markers for early endosomes (Rab5a), late endosomes (Rab7a) or lysosomes (Lamp1). The fraction of organelle in which colocalization of APP/BACE1 occurred was calculated as percentage of the area of PLA/organelle colocalization against the area of total organelle (see methods for details). Since there was no significant difference in total organelle area of each organelle between NPCs and neurons (supplementary Fig. 1C), the change in PLA/organelle colocalization was only dependent on the PLA dots area and location. In neurons compared to NPCs, the colocalization of BACE1 with flAPP was significantly increased in early endosomes and decreased in late endosomes (Fig. 3J), suggesting that early endosomes are the preferred endocytic site of β-site cleavage of APP in neurons. In neurons compared to NPCs, the colocalization of BACE1 with C-terminal APP was significantly decreased in early endosomes and lysosomes, with a tendency to decrease in late endosomes (Fig. 3K), in line with the overall decrease in total C-terminal APP/BACE1 colocalization.

Interestingly, when investigating the protein levels of both direct products of BACE1 cleavage, sAPPβ and CTFβ, we found that the levels of secreted sAPPβ were increased about 20-fold in neurons as compared to NPCs (Fig. 3L), whereas no difference in intracellular levels of CTFβ was found (Fig. 3M). This suggests that CTFβ is rapidly cleaved to Aβ in neurons, whereas it retains the interaction with BACE1 in NPCs.

### Colocalization of PSEN1 with APP-CTF correlates with Aβ Secretion

**Fig. 4:**
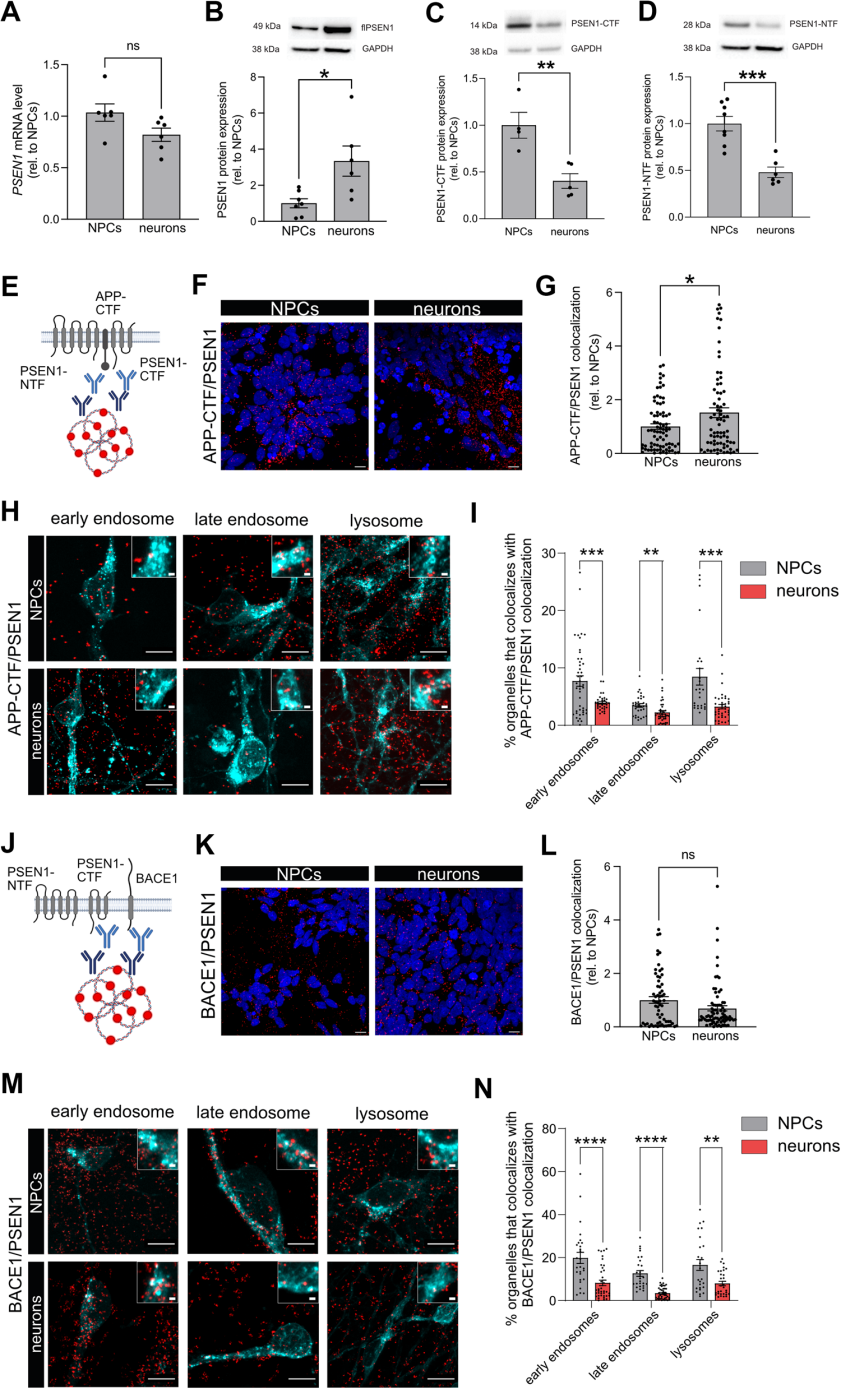
Aβ secretion correlates with APP-CTF/PSEN1 colocalization but not BACE1/PSEN1 colocalization. **A** *PSEN1* mRNA was investigated using qPCR. No difference in *PSEN1* mRNA was detected between NPCs and neurons. Bars represent mean of three separate differentiations from two different iPSC lines ± SEM. **B**–**D** Protein expression of full-length PSEN1 and catalytically active fragments PSEN1-CTF and -NTF were investigated using western blot. A significant increase in PSEN1 protein expression, but a significant decrease in both PSEN1 active fragments were detected in neurons as compared to NPC. Full-length blots are available in Supplementary Fig. 2. Bars represent mean of six separate differentiations from two different iPSC lines ± SEM. **E**–**G** A proximity ligation-based immunocytochemistry assay was used to investigate the colocalization of PSEN1 with the C-terminal fragment of APP (APP-CTF). **E** A schematic image of the binding sites for the oligonucleotide-tagged antibodies detecting PSEN1 and APP-CTF. **F** Colocalization, visualized as red dots, between PSEN1 and APP-CTF was increased in neurons as compared to NPCs. Scale bar = 10 μM. **G** A significant increase in colocalization signal per cell was found in neurons as compared to NPCs. Data are presented as fold change related to NPCs, set to 1 and bars represent mean + / − SEM. 79 (NPCs) or 74 (neurons) images from four to five separate experiments on cells from at least two separate differentiations were analysed, **H** Signal overlap between APP-CTF/PSEN1 PLA (red) and GFP-tagged organelle markers for early endosomes (Rab5a) late endosomes (Rab7a) and lysosomes (lamp1) (cyan) was used to determine intracellular localization of APP-CTF/PSEN1 colocalization. Smaller boxes show a zoom-in of organelle colocalization with PLA signals. Scale bar = 10 μm (large boxes), 1 μm (small boxes). **I** A significant decrease in colocalization of APP-CTF/PSEN1 was found in neurons as compared to NPCs in all endocytic organelles. Data are presented as percentage of total organelle area and bars represent mean + / − SEM. 26–45 images for each organelle from three separate experiments on cells differentiated from two different iPSCs lines were analysed. **J-L** A proximity ligation-based assay was used to investigate the colocalization of BACE1 with PSEN1. **J** A schematic image of the binding sites for the oligonucleotide-tagged antibodies detecting BACE1 and PSEN1. **K** Colocalization, visualized as red dots, between BACE1 and PSEN1 was detected both in NPCs and neurons. Scale bar = 10 μm. **L** When quantified, no significant differences in colocalization signal per cell was found, although a tendency to decreased colocalization could be seen in neurons as compared to NPCs. Data are presented as fold change related to NPCs, set to 1 and bars represent mean + / − SEM. 63 (NPCs) or 78 (neurons) images from five to six separate experiments on cells from three separate differentiations were analysed. **M** Signal overlap between BACE1/PSEN1 PLA (red) and GFP-tagged organelle markers for early endosomes (Rab5a) late endosomes (Rab7a) and lysosomes (lamp1) (cyan) was used to determine intracellular localization of BACE1/PSEN1 colocalization. Smaller boxes show a zoom-in of organelle colocalization with PLA signals. Scale bar = 10 μm (large boxes), 1 μm (small boxes). **N** A significant decrease in all organelles colocalization with BACE1/PSEN1 was found in neurons as compared to NPCs. Data are presented as percentage of total organelle area and bars represent mean + / − SEM. 25–44 images for each organelle from three separate experiments on cells differentiated from two different iPSCs lines were analysed. All mean comparisons were done using Student’s *t* test. *p ≤ 0.05, **p ≤ 0.01, ***p ≤ 0.001, ****p ≤ 0.0001

Generation of Aβ requires a second cleavage of the CTFβ by γ-secretase, a multi-protein complex with proteolytic activity. The active subunit of this γ-secretase complex is PSEN1, and we therefore examined the expression of PSEN1 mRNA and protein. *PSEN1* mRNA expression did not differ between NPCs and neurons (Fig. 4A). PSEN1 holoprotein expression was significantly increased about threefold (Fig. 4B), while both PSEN1-CTF and -NTF protein expression was significantly decreased in high-Aβ producing neurons as compared to NPCs (Fig. 4C–D). To explain this inconsistency, we investigated the colocalization of PSEN1 with APP-CTF, using the PLA (Fig. 4E). Both CTFα and CTFβ can interact with PSEN1, and the C-terminal APP antibody used here does not discriminate between the two CTF forms. Colocalization of APP-CTF and PSEN1 was significantly increased in neurons as compared to NPCs, in line with the increased secretion of amyloidogenic Aβ peptides (Fig. 4E–G). This may explain why the levels of CTFβ remained stable in the cortical neurons despite higher β-secretase cleavage. Since endosomes and lysosomes are proposed sites for γ-secretase cleavage of APP and Aβ production (Maesako et al. 2022; Dries and Yu 2008), we investigated the colocalization of APP-CTF/PSEN1 in early endosomes, late endosomes and lysosomes (Fig. 4H–I) and found that APP-CTF/PSEN1 colocalized to a lower degree in neurons as compared to NPCs in all three organelles.

### Colocalization of BACE1 with PSEN1 in Endo-Lysosomal Organelles Does Not Correlate with Aβ Production

BACE1 and PSEN1 were recently reported to work together in a multicomplex that accounted for most of the production of Aβ (Liu et al. 2019). Therefore, we used PLA to investigate the proximity of the two enzymes in our cell model using antibodies binding at the C-terminus of both proteins (Fig. 4J). Indeed, PLA signals were detected in both NPCs and neurons (Fig. 4K) but there were no significant differences between the two (Fig. 4L). This may suggest that BACE1/PSEN colocalization does not regulate the rate of Aβ production in neurons. In addition, the percentage of all three organelles colocalizing with the BACE1/PSEN1 complex was significantly decreased in neurons compared to NPCs (Fig. 4M, N). This may suggest other roles for BACE1/PSEN1 colocalization than to regulate the rate of Aβ production in neurons.

## Discussion

Only a handful of studies have investigated the direct physical interaction of APP and β- or γ-secretase in human neuronal cell models, and most of them have used transient transfection of fluorescent-labelled proteins to closely follow APP/secretases interaction during the whole of APP processing (Kinoshita et al. 2003; Nizzari et al. 2007; Das et al. 2013, 2016). Fluorescent labels bear intrinsic limitations, connected to their high molecular weight that can disturb the protein structure, function, localization and stability (Jensen 2012). Protein overexpression itself can alter APP or secretase localization and turnover, as recent work has demonstrated (Aow et al. 2022). This highlights the need for new tools to study APP/secretases interaction without altering their trafficking. An alternative approach is to compare models of lower vs higher Aβ secretion. Increased Aβ secretion has been achieved either by pharmacological treatments (Das et al. 2013) or introduction of mutated variants of APP or the secretases (Takeda et al. 2004). However, there is always a risk that such manipulations also impact the APP or secretase localization and/or turnover.

We here performed a comparative study of two genetically identical neuronal cell types in which increased Aβ secretion is achieved physiologically along with neuronal maturation. This model system has two important advantages: (1) intracellular and secreted levels of substrate (APP), enzymes (BACE1, PSEN1) and products (sAPPβ, CTFβ, Aβ40/42) of APP amyloidogenic processing can be measured and correlated to the colocalization of APP and β-/γ-secretase; (2) comparison between all these factors can be performed when the cells physiologically shift from low to high secretion of Aβ, without the need of genetically or pharmacologically modifying any of the factors involved in APP processing.

Another interesting aspect of this model is that NPCs and neurons, by being in separate differentiation stages, are two distinct cell types. Both express all the known basic components necessary for Aβ production, yet neurons show a much higher rate of amyloidogenic APP cleavage. Here, APP protein expression was significantly increased in neurons whereas an uneven fluctuation of APP protein expression throughout neuronal differentiation was detected in our previous study (Bergstrom et al. 2016). Therefore, we hypothesize that APP protein increase in neurons compared to NPCs likely reflects cell line-specific differences rather than a mechanism for physiological increased Aβ production. Indeed, no difference in BACE1 and even decreased PSEN1-NTF and -CTF protein expression was found in neurons compared to NPCs. This emphasizes that increased APP amyloidogenic processing does not rely on substrate and enzyme protein expression alone. APP and BACE1 intracellular trafficking, in particular, is a highly dynamic process, involving multiple subcellular localizations from the endoplasmic reticulum to the plasma membrane and endocytic compartments (Zhang and Song 2013).

An important mechanism for the regulation of APP processing is the physical interaction of APP with β- and γ-secretases (Das et al. 2016). Here, we investigated the colocalization of APP with the secretases using PLA, an immunocytochemistry method that allows high resolution visualization of protein proximity, which has been previously used to determine the secretases intracellular localization (Lundgren et al. 2020) and novel secretases-interacting proteins (Teranishi et al. 2015).

The two relevant membrane-bound APP species for the production of Aβ are flAPP, substrate of BACE1, and APP-CTFβ, product of BACE1 and substrate of PSEN1. We distinguished between the two by using antibodies directed towards epitopes at different ends of the protein. While the C-terminal end is shared by both flAPP and APP-CTF, the N-terminal is exclusively found in flAPP, as it is shed off by the first cleavage.

Increased colocalization of flAPP with BACE1 in mature neurons, together with increased production of sAPPβ in these cells indicates that proximity of flAPP and BACE1 positively regulates APP cleavage. This shift in colocalization most likely depends on convergence in the same subcellular organelle, as several studies in neurons and non-neuronal cells have suggested (reviewed in Zhang and Song 2013). Indeed, we found that colocalization of flAPP with BACE1 increased in early endosomes, but decreased in late endosomes in neurons. These results suggest that early endosomes may be the major endo-lysosomal site of BACE1 cleavage of APP, which is in line with previous findings (Kinoshita et al. 2003; Toh et al. 2017, 2018).

In neurons, alternative cleavage of flAPP is carried out by α-secretases, such as ADAM10 and ADAM17, in a separate non-amyloidogenic pathway (Portelius et al. 2011). Although the α- and β-secretase pathways have been shown to be only partially coupled in neurons, with ADAM10 compensating for the pharmacological inhibition of BACE1 (May et al. 2011; Colombo et al. 2013), we cannot exclude that α-secretase/APP interaction is involved in the regulation of Aβ production in our comparative model and this should be further studied.

Opposite to flAPP, the colocalization of C-terminal APP with BACE1 was decreased by almost 80% in neurons. As the colocalization between BACE1 and N-terminal APP was increased by 63% in mature neurons, we calculated that the decrease in colocalization between BACE1 and C-terminal APP mainly originates from the CTF and that APP-CTF/BACE1 colocalization is at least 4.7 times less in neurons than in NPCs (see supplementary methods). This indicates a faster product/enzyme complex dissociation in neurons secreting 20 times more sAPPβ.

More unexpectedly, APP-CTF/BACE1 colocalization was calculated to be at least 6.7 times greater than flAPP/BACE1 colocalization in NPCs secreting considerably lower levels of both sAPPβ and Aβ (see supplementary methods). Thus, CTF colocalization with BACE1 seems to negatively regulate BACE1 activity and/or CTF processing. This could be explained in several ways. First, retention of CTFβ in the proximity of BACE1 might have an inhibitory feedback effect on its own production by regulating BACE1 activity. Several studies have shown that APP, both in its full-length or cleaved forms, can regulate its own processing by modulating the activity of the cleaving enzymes (Lesné et al. 2005; Tian et al. 2010a; Obregon et al. 2012; Beckmann et al. 2016). For example, two out of three major APP isoforms can inhibit the α-secretase ADAM17 through the so called Kunitz-type protease inhibitor (KPI) domain (Lesné et al. 2005; Kuhn et al. 2010), thereby shifting from α- to β-secretase APP processing. In addition, CTFα, the transmembrane APP fragment produced by α-secretase cleavage is predicted to bind the γ-secretase complex allosterically, inhibiting APP γ-cleavage (Tian et al. 2010a, b). Soluble APPα has also been shown to have direct inhibitory interaction with β-secretase (Obregon et al. 2012). However, to the best of our knowledge, no inhibition of β-secretase by flAPP or CTF has been reported so far. It has to be noted that the use of a C-terminal antibody for APP does not exclude binding of CTFα, which is not a known substrate of BACE1 but could also be interacting in an allosteric manner to modulate BACE1 activity. Another possible reason for BACE1-CTFβ colocalization in NPCs, is that BACE1 could function as a modulator and prevent physical interaction between PSEN1 and CTFβ in NPCs. This would stop biosynthesis and secretion of Aβ as well as prevent potential known cytotoxicity from the unprocessed CTFβ (Oster-Granite et al. 1996; Yankner Bruce et al. 1989; Lauritzen et al. 2016; Vaillant-Beuchot et al. 2021). Indeed, the higher colocalization of APP-CTF with BACE1 in all three organelles in NPCs could reflect the routing of a stabilized inhibitory CTF/BACE1 complex directed to lysosomal degradation.

Despite higher β-cleavage of APP, intracellular levels of CTFβ did not differ between NPCs and neurons. This likely reflects a rapid cleavage of CTFβ serving as a substrate for γ-secretase. Indeed, APP-CTF/PSEN1 colocalization was overall increased in neurons together with Aβ secretion. However, APP-CTF/PSEN1 colocalization was decreased in all stages of endosomes maturation towards lysosomes. This is in contradiction to earlier reports where PSEN1 activity is mainly found in late endosomes and lysosomes (Pasternak et al. 2004; Maesako et al. 2022). However, the subcellular localization of γ-secretase activity in relation to APP processing is still under debate and other cellular locations, such as plasma membrane and lipid raft (Vetrivel et al. 2005; Ehehalt et al. 2003; Chyung et al. 2005), have been shown to be additional cleaving γ-sites for APP.

In addition to PSEN1, PSEN2 can also act as the catalytic subunit in the γ-secretase complex (Zhang et al. 2000; Meckler and Checler 2016), but PSEN1 has been the main focus in studies regarding neuronal Aβ production, since mutations in *PSEN1* lead to earlier onset and clinically more severe AD compared to *PSEN2* (Kabir et al. 2020; Herreman et al. 1999). However, PSEN2-containing γ-secretase complexes are also active in Aβ production and it has been reported that PSEN2 is enriched in late endosomes and lysosomes and is specifically responsible for the generation of an intracellular Aβ pool (Meckler and Checler 2016; Sannerud et al. 2016). Future studies are needed to investigate how APP-CTF and PSEN2 interaction and localization affects Aβ production and secretion.

More recently, an alternative model for Aβ generation was proposed, where BACE and γ-secretase were suggested to work together in a multicomplex that accounted for most of the production of Aβ (Liu et al. 2019). Although we could detect colocalization of BACE1 and PSEN1 in both NPCs and neurons, the interaction did not change significantly between the two cell types and their colocalization in all endocytic organelles was significantly decreased in neurons. Although confirming the presence and trafficking of a BACE/γ-secretase complex in human neurons, this does not seem to directly regulate Aβ generation.

Taken together, we present an in vitro comparative model where processes involved in Aβ generation can be studied in a manipulation-free manner in high Aβ-secreting mature neurons and low Aβ-secreting NPCs. We demonstrate that mature neurons show increased colocalization of flAPP with BACE1 in early endosomes and increased colocalization of APP-CTF with PSEN1, although the subcellular location was not established. BACE1 cleavage seems to be the rate-limiting step for Aβ production, since all CTFβ produced is cleaved into Aβ in neurons. NPCs, on the other hand, showed a strong interaction of CTF with BACE1, suggesting a negative CTF-BACE1 regulatory mechanism for Aβ production.

One limitation of the PLA method is that it is an end-point technique, therefore it can only address the steady-state interaction and localization of the proteins involved in Aβ generation. Therefore, comparing these findings with live-tracking of APP/secretases interaction (e.g. using fluorescent-labelled proteins) will further shed light on the mechanisms of APP processing.

Regulation of APP-secretases interaction in neurons is an unresolved question in AD biology. The proposed interaction of APP-CTF with BACE1 as a negative cellular mechanism for Aβ production, could open up for new preventive strategies to AD pathogenesis.

## Supplementary Information

Below is the link to the electronic supplementary material.

Supplementary file1 (DOCX 4949 KB)

## Data Availability

The datasets used and/or analysed during the current study are available from the corresponding author on reasonable request.
