# Supplementary material for: APP-BACE1 Interaction and Intracellular Localization Regulate Aβ Production in iPSC-Derived Cortical Neurons
Source: Cell Mol Neurobiol. 2023 Jun 24;43(7):3653–68. doi: 10.1007/s10571-023-01374-0 (PMC10477112; doi:10.1007/s10571-023-01374-0)
Supplement: Supplementary file 1 — Supplementary file1 (DOCX 4949 KB) [file 10571_2023_1374_MOESM1_ESM.docx]

**Supplementary information**

**APP-BACE1 interaction and intracellular localization regulate Aβ production in iPSC-derived cortical neurons**

Sandra Roselli^a§*^ (ORCID ID: 0000-0003-2998-3760), Tugce Munise Satir^a§^ (ORCID ID: 0000-0001-9021-1650), Rafael Camacho^b^ (ORCID ID: 0000-0003-2325-6407), Stefanie Fruhwürth^a^ (ORCID ID: 0000-0003-4035-7330), Petra Bergström^a#^ (ORCID ID: 0000-0003-1803-165X), Henrik Zetterberg^a,c,d,e,f,g#^ (ORCID ID: 0000-0003-3930-4354), Lotta Agholme^a#^ (ORCID ID: 0000-0003-3816-7474)

1. *Institute of Neuroscience and Physiology, Department of Psychiatry and Neurochemistry, the Sahlgrenska Academy at the University of Gothenburg, Blå stråket 15, Sahlgrenska Hospital, Gothenburg, S-405 30, Sweden*
2. *Centre for Cellular Imaging, Core Facilities, The Sahlgrenska Academy, University of Gothenburg, Medicinaregatan 7A, Gothenburg, S-405 30, Sweden*
3. *Clinical Neurochemistry Laboratory, Sahlgrenska University Hospital, Building V3, Mölndal Hospital, Mölndal, S-431 80, Sweden*
4. *Department of Neurodegenerative Disease, Institute of Neurology, University College London Queen Square, Queen Square, London, WC1N 3BG, UK*
5. *UK Dementia Research Institute at UCL,* *Cruciform Building, Gower Street, London, WC1E 6BT, UK*
6. *Hong Kong Center for Neurodegenerative Diseases, Units 1501-1502, 1512-1518, 15/F, Building 17W, Hong Kong Science Park, Shatin, N.T., Hong Kong, China*
7. *Wisconsin Alzheimer’s Disease Research Center, University of Wisconsin School of Medicine and Public Health, University of Wisconsin-Madison, 600 Highland Avenue, Madison, WI 53792, USA*

§, #Equal contribution

*^*^*Corresponding author

Institute of Neuroscience and Physiology, Department of Psychiatry and Neurochemistry, the Sahlgrenska Academy at the University of Gothenburg, Blå stråket 15, Sahlgrenska Hospital, Gothenburg, S-405 30, Sweden

Phone: +46 (0)31 786 1000 (main switchboard)

Fax: +46 (0)31 343 24 26

Email address: [sandra.roselli@gu.se](mailto:sandra.roselli@gu.se) (S. Roselli)

**Supplementary methods.**

**System of equations and inequalities to describe the relation of APP-CTF/BACE1 colocalization between NPCs and neurons.**

Assumptions:

1. N-terminal APP/BACE1 colocalization is equal to flAPP colocalization
2. C-terminal APP/BACE1 colocalization is a sum of APP-CTF/BACE1 colocalization and flAPP/BACE1 colocalization
3. Absolut values of colocalization of N-terminal and C-terminal APP with BACE1 cannot be directly compared because of unknown differences in the antibody affinity for the target proteins. However, the ratio of colocalization of each APP species and BACE1 between NPCs and neurons should be independent of antibody affinity. Therefore, only ratios will be included in the relation between variables.

Variables:

*x1*:= mean flAPP/BACE1 colocalization in NPCs

*x2*:= mean APP-CTF/BACE1 colocalization in NPCs

*y1*:= mean flAPP/BACE1 colocalization in neurons

*y2*:= mean APP-CTF/BACE1 colocalization in neurons

System of equations and inequalities:

$$y1=1.62x1$$

$$y1 + y2 = 0.21\left( x1 + x2 \right)$$

$$x1, y2\geq0$$

Relation 1:

$$x2\geq4.7y2$$

Therefore, the mean APP-CTF/BACE1 colocalization is at least 4.7 times smaller in neurons than in NPCs.

Relation 2:

$$x2\geq6.7x1$$

Therefore, the mean APP-CTF/BACE1 colocalization in NPCs is at least 6.7 times greater than the mean flAPP/BACE1 colocalization in NPCs.

**
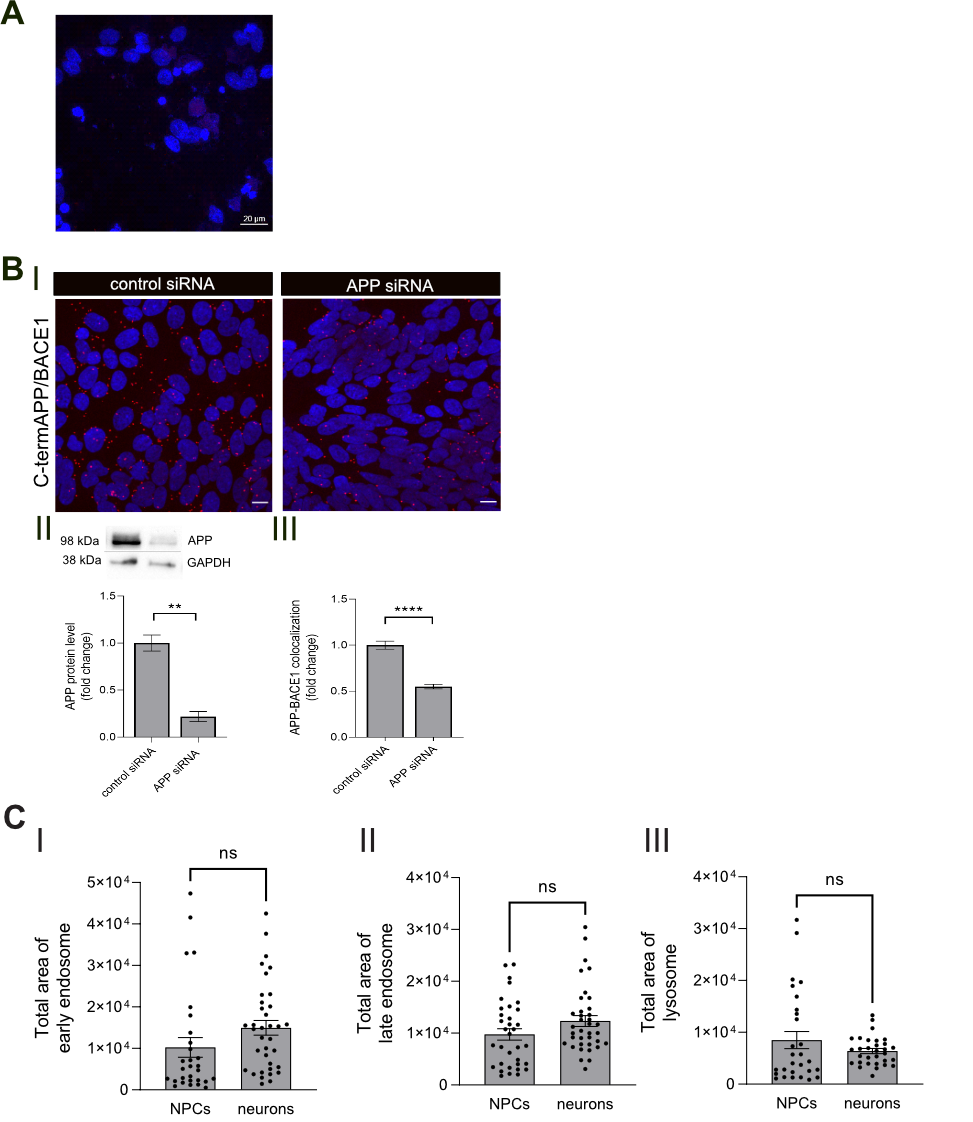
**

**Supplementary figure 1. PLA control experiments.** A) Representative images of PLA technical control. Neurons with high Aβ secretion were incubated with antibody directed towards APP only and subjected to PLA. This negative control experiment did not generate any signals supporting the specificity of the assay. B) PLA control experiment using gene knockdown in SH-SY5Y cells. Protein expression of amyloid precursor protein (APP) was knocked down in SH-SY5Y cells using APP-small interfering RNA (siRNA); protein levels of APP for control and APP knockdown cells were measured using western blot, and the colocalization of APP (C-terminal antibody) with BACE1 was investigated, using PLA. (i) Representative images of red DuoLink dots representing APP‒BACE1 colocalization in cells transfected with control siRNA or APP siRNA, respectively. Scale bar = 10 μm. (ii) Intracellular APP protein levels are significantly decreased in cells transfected with APP siRNA compared to control siRNA. Bars represent mean of three separate experiments +/- SEM, **p ≤ 0.01. (iii) Relative quantification of the APP‒BACE1 colocalization dots showed decreased APP‒BACE1 interaction in APP knockdown SH-SY5Y cells compared to control. Thirty-six images from three separate experiments were analysed with Student’s t-test., ****p ≤ 0.0001. Bars represent mean +/− SEM. C) Control experiment for intracellular localization of APP/secretases colocalization. Total area of GFP-tagged (i) Rab5a (early endosome), (ii) Rab7a (late endosome) and (iii) lamp1 (lysosome) did not differ significantly between NPCs and neurons, showing that changes in % of organelle occupied by PLA dots only depends on PLA dots area and localization. Bars represent mean +/− SEM of three separate experiments with two iPSC lines.

**
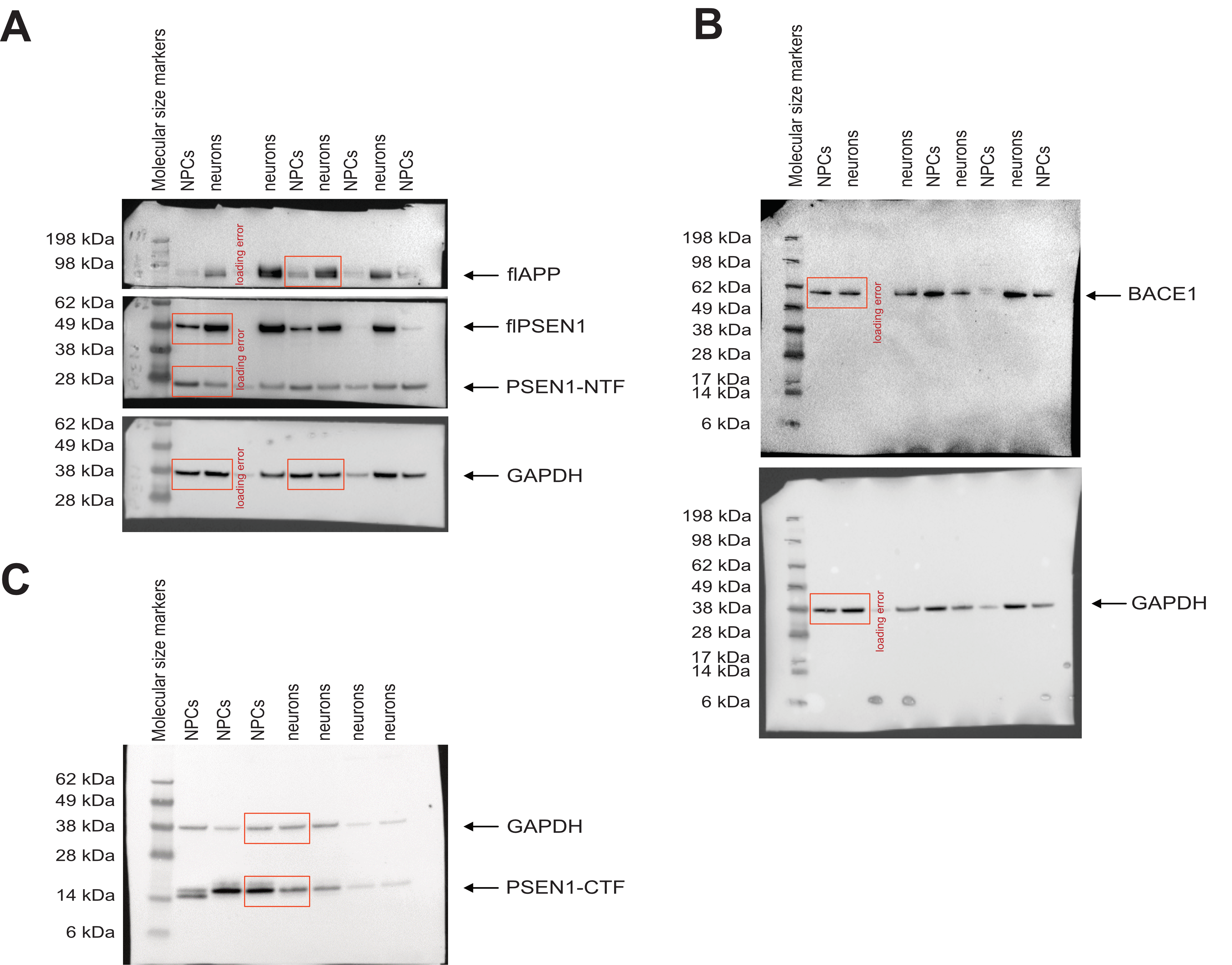
**

**Supplementary figure 2. Representative images of whole membrane blots.**

A) Representative blots of APP (upper membrane), PSEN1 (middle membrane) and housekeeping protein GAPDH (lower membrane). The arrow points out the full-length APP band (~98 kDa), PSEN1 band (~49 kDa), PSEN1-NTF band (~28 kDa), and GAPDH band (~38 kDa). B) Representative blot of BACE1 (upper membrane) and housekeeping protein GAPDH (lower membrane). The arrow points out the BACE1 band (~62 kDa) and the GAPDH band (~38 kDa). The sample in lane 4 was excluded from the calculations in both blots, due to a loading error. C) Representative blot of PSEN1-CTF and housekeeping protein GAPDH. The arrow points out the BACE1 band (~14 kDa) and the GAPDH band (~38 kDa). The red framed bands are the ones shown in the figures for APP, BACE1 and PSEN1 and respective loading control GAPDH.

| Target Protein | Host | Company | Catalogue number | Dilution |
| --- | --- | --- | --- | --- |
| Anti-β-Amyloid, 1-16 Antibody | Mouse | Biolegend | 803002 | WB 1:1000 |
| BACE1 | Mouse | Sigma Aldrich | MAB5308 | WB 1:1000  PLA 1:200 |
| GAPDH HRP conjugated | Mouse | Novus biologicals | NB300-328H | WB 1:30000 |
| MAP2 | Mouse | Abcam | ab11267 | ICC 1:500 |
| Presenilin-1 (NH2-terminus) | Rabbit | Biolegend | 811101 | WB 1:1000  PLA 1:100 |
| Presenilin-1 (C-terminus) | Mouse | Sigma Aldrich | MAB5232 | PLA 1:200  WB 1:1000 |
| Tau | Chicken / IgY | Biorbyt | orb175815 | ICC 1:1000 |
| APP-C terminal | Rabbit | Sigma Aldrich | 171610 | PLA 1:500 |
| APP-N terminal | Rabbit | Abcam | ab15272 | PLA 1:500 |
| Nestin | Mouse | Millipore | MAB1259 | ICC 1:50 |
| PAX6 | Rabbit | Biolegend | PRB-278P | ICC 1:500 |
| TBR1 | Rabbit | Abcam | ab31940 | ICC 1:250 |
| goat anti- mouse Alexa488 |  | Thermo Fisher Scientific | A11001 | ICC 1:400 |
| goat anti-chicken Alexa488 |  | Thermo Fisher Scientific | A11056 | ICC 1:400 |
| goat Anti-mouse Alexa568 |  | Thermo Fisher Scientific | ab175473 | ICC 1:400 |
| goat anti-rabbit Alexa488 |  | Thermo Fisher Scientific | A11070 | ICC 1:400 |
| goat Anti-mouse Alexa647 |  | Thermo Fisher Scientific | A28181 | ICC 1:400 |
| goat Anti-rat Alexa647 |  | Thermo Fisher Scientific | A21247 | ICC 1:400 |
| goat Anti-rabbit Alexa568 |  | Thermo Fisher Scientific | A11011 | ICC 1:400 |

**Supplementary table 1. Antibodies and concentrations used in western blot, immunocytochemistry and proximity ligation assay.**

| Gene Name | Company | Catalogue number |
| --- | --- | --- |
| Amyloid beta precursor protein (APP) | Thermo Fisher Scientific | Hs00169098_m1 |
| B-cell CLL/lymphoma 11B (CTIP2/BCL11B) | Thermo Fisher Scientific | Hs01102259_m1 |
| Beta-secretase 1 (BACE1) | Thermo Fisher Scientific | Hs01121195_m1 |
| Hypoxanthine phosphoribosyl transferase 1 (HPRT1) | Thermo Fisher Scientific | Hs02800695_m1 |
| Paired Box 6 (PAX6) | Thermo Fisher Scientific | Hs00240871_m1 |
| POU class 3 homeobox 2 (BRN2) | Thermo Fisher Scientific | Hs00271595_s1 |
| Presenilin 1 (PSEN1) | Thermo Fisher Scientific | Hs00997789_m1 |
| Ribosomal protein L30 (RPL30) | Thermo Fisher Scientific | Hs00265497_m1 |

**Supplementary table 2. Primers used for qPCR**
